# Perioperative Complications in Children with Down Syndrome: A Single Center Retrospective Analysis—Original Clinical Research Report

**DOI:** 10.3390/jcm14092900

**Published:** 2025-04-23

**Authors:** Michelle Tsao, Frank Yanko, Eric Cheon

**Affiliations:** Department of Pediatric Anesthesiology, Ann & Robert H. Lurie Children’s Hospital of Chicago, Feinberg School of Medicine, Northwestern University, Chicago, IL 60611, USA; frank.yanko@northwestern.edu (F.Y.); echeon@luriechildrens.org (E.C.)

**Keywords:** down syndrome, trisomy 21, pediatrics, anesthesia, perioperative outcomes, complications

## Abstract

**Background/Objectives:** Down syndrome (DS) is the most common chromosomal abnormality in live births in the United States. Children with DS often require anesthesia for surgery or diagnostic imaging in their lives. These children present a unique perioperative risk profile due to a combination of anatomic and physiological alterations, along with associated comorbid conditions. There are limited studies on the perioperative outcomes of children with DS. This retrospective study assesses perioperative complications in pediatric patients with DS undergoing non-cardiac surgery or diagnostic imaging under anesthesia at a single tertiary pediatric hospital. **Methods:** The electronic medical record at a tertiary pediatric hospital was queried for children with DS who received anesthesia for non-cardiac surgery or diagnostic imaging from May 2016 to April 2021. The primary outcomes were complications defined as readmission, reoperation, or unexpected respiratory, cardiovascular, neurologic, surgical, or gastrointestinal issues. Exclusion criteria were cardiac surgery, age > 18 years, and records with incomplete or missing data. **Results:** A total of 1713 anesthetic records from 711 unique patients over five years were included in the final analysis. The study found a low overall complication rate (2.98%), with respiratory events being the most common (43.1%). While most complications are short term and resolved with treatment and time; there were also several severe, life-threatening complications. Increased procedural complexity, multiple procedures, and increased procedure duration were associated with higher complication rates, whereas patient age, sex, weight, and case urgency were not associated with higher complication rates. **Conclusions:** Children with DS often have comorbid conditions and require multiple life-improving surgeries. Our study found the perioperative complication rate for children with Down syndrome receiving anesthesia for non-cardiac surgery or diagnostic imaging is low, comparable to the general pediatric population. The findings indicate that anesthesia is well tolerated by children with DS. However, given patients’ unique anatomic and physiological differences, careful perioperative risk assessment and planning is essential. Clinical Implications: (a) What is already known about the topic: Pediatric patients with DS often require anesthesia for surgical procedures or medical imaging. They have anatomic and physiological alterations and comorbid conditions that may influence perioperative risk. (b) What new information this study adds: In a retrospective study at a tertiary pediatric hospital, patients with DS were found to have a low overall complication rate after anesthesia for non-cardiac surgery or diagnostic imaging. Increased procedural complexity, multiple procedures, and increased procedure duration were associated with higher complication rates.

## 1. Introduction

Down syndrome (DS) is the most common chromosomal abnormality in live births in the United States occurring at a rate of approximately 1 in 1000 births [1]. The French physicians Jérôme Lejeune, Gautier, and Turpin first described the association between DS and a third chromosome 21 in 1959 [2]. Since the 1950s, advances in healthcare have greatly improved life expectancy for individuals with DS. Estimated survival in the first year of life has increased from 46–71% in the 1950s [3,4] to 91% in the 1990s [5]. Life expectancy has increased to 53–60 years in the 2000–2010s [6,7]. The increase in life expectancy is attributable to advances in corrective cardiac surgery and overall improved healthcare [8].

Children with DS often require anesthesia for surgery or diagnostic imaging throughout their lives. As individuals with DS live longer, an increase in the number of anesthetic encounters is likely. These children present a unique perioperative risk profile due to a combination of anatomic and physiological alterations, along with associated comorbid conditions. Airway abnormalities including macroglossia, glossoptosis, subglottic stenosis, and atlantoaxial instability may contribute to increased difficulty with airway management [9,10,11,12,13,14,15,16]. Airway obstruction is very common and exacerbated by adenoid and tonsillar hypertrophy, a higher prevalence of obesity, and high incidence of obstructive sleep apnea [17,18,19,20,21]. Additionally, congenital heart disease is present in approximately 40% of children with DS [22,23] and certain specific variants are associated increased mortality as compared to children without DS [24]. Other health concerns that influence perioperative management include sensitivity to anesthetic agents [25,26], gastrointestinal abnormalities [22,27,28], impaired immune response [29], endocrine disorders [12,27], hematologic conditions [12,27], and intellectual disability affecting patient understanding and cooperation [12,27].

While large, prospective, multicenter studies have reported perioperative outcomes in the general pediatric population [30,31,32,33,34], data specific to children with DS remain limited. Much of the existing research is retrospective, small, and focused on a specific subset of procedures [35,36,37,38,39]. Some studies report no increased complication rates [36], while others suggest a higher risk [40]. Consequently, there is a need for updated, comprehensive research on perioperative risks and outcomes in this unique population across a broad range of surgical and imaging procedures. This is relevant to both general and pediatric anesthesiologists since greater than 80% pediatric surgeries are performed as ambulatory surgery [41]. The success of ambulatory surgery depends on careful selection, screening, and optimization [42,43]. It is important to understand perioperative complications in patients with DS to develop strategies to minimize complications and improve care.

The present study aims to address this gap by reporting on the incidence and types of perioperative complications in children with DS undergoing anesthesia at a single pediatric tertiary care center. Specifically, we seek to identify the most common perioperative adverse events and analyze factors associated with these complications.

## 2. Methods

The Ann and Robert H. Lurie Children’s Hospital of Chicago Institutional Review Board deemed this study as exempt from review, with a waiver of signed patient consent. This was a single center retrospective cohort study of patients with DS. A chart review of all anesthetics occurring in patients with DS between May 2016 and April 2021 was performedusing the Epic Systems electronic medical records platform (Verona, WI, USA) and a dataset was created with the following information: age, patient weight, gender, American Society of Anesthesiologists (ASA) physical status, surgical specialty, relative value units (Centers for Medicare and Medicaid Services Resource Based Relative Value Scale or RVUs), length of case, length of stay, complications, procedures performed, and urgence of case booking. Work RVUs [44] were summed based on the procedures recorded. Work RVUs were used as a proxy for case complexity [45]. Each anesthetic record was reviewed for perioperative adverse events by one of the authors. Complications were defined as readmission, reoperation, or unexpected respiratory, cardiovascular, neurologic, surgical, or gastrointestinal issues. Exclusion criteria were cardiac surgery, age > 18 years, and records with incomplete or missing data. Continuous variables are presented as mean (standard deviation) for normally distributed data or median [interquartile range] for non-normal distributions. Categorical variables are reported as frequencies (percentages). Group comparisons (complications vs. no complications) were performed using independent *t*-tests or Wilcoxon rank-sum tests for continuous variables and chi-square or Fisher’s exact tests for categorical variables, as appropriate. Two-sided *p*-values < 0.05 were considered statistically significant. A univariate logistic regression model was used to assess the association between each characteristic and complications. Surgical specialties that did not have complications were excluded from the logistic regression model because calculating an odds ratio was not possible. Due to collinearity between the covariates and specialties, we could only adjust for age and gender in the multivariate logistic regression model. In this, the univariate model is sufficient, as age and gender are not associated with specialty line in the results. All statistical analyses were conducted using R version 4.4.0 (R Foundation for Statistical Computing, Vienna, Austria). As this was a descriptive study where the primary goal is to describe and summarize data without hypothesis testing, a formal power analysis was not performed.

## 3. Results

**Figure 1:**
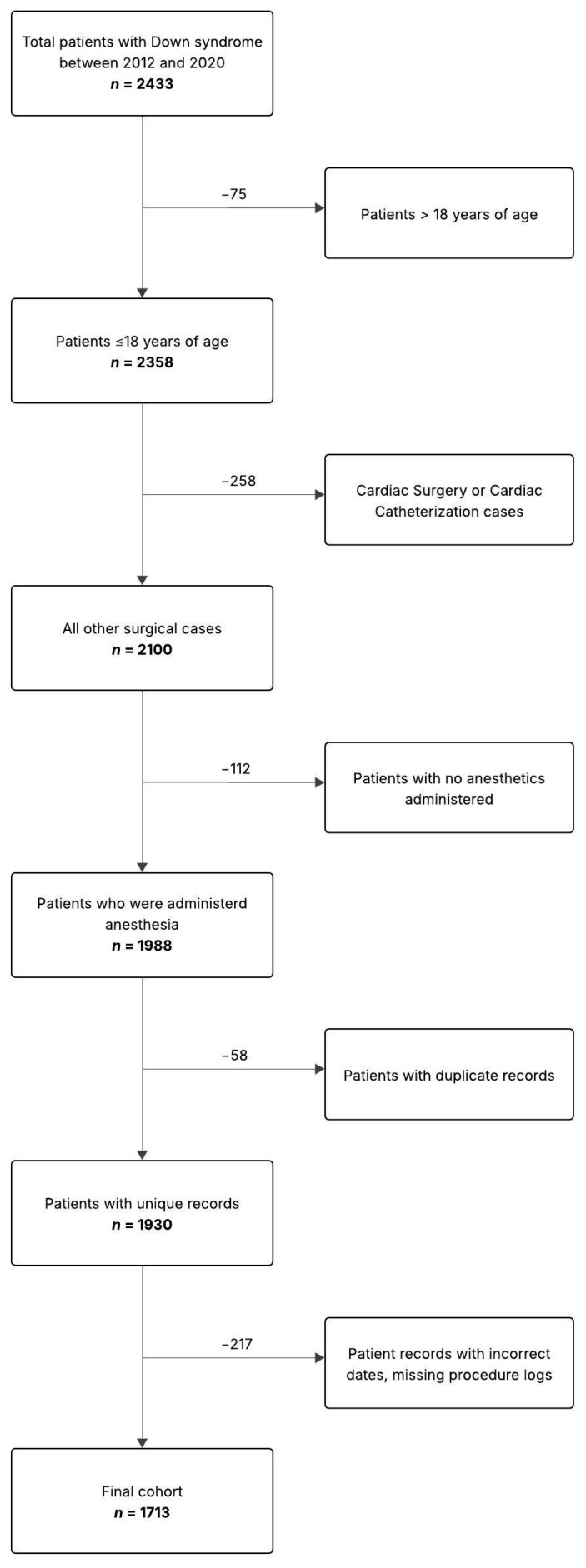
Patient flow diagram.

The final cohort consisted of 1713 anesthetic procedures with 711 unique patients that met the inclusion criteria (Figure 1). Of these 711 unique patients, 366 patients (51.5%) received a single anesthetic, while 345 (48.5%) patients received two or more anesthetics over the study period. Thirty-five cases (2.0%) were performed with sedation while the remainder were performed under a general anesthetic. There were 380 planned admissions (22%), 293 in-patient encounters (17%), 1029 same-day discharge encounters (60%), and 11 unanticipated admissions (0.64%). Table 1 lists the characteristics of patients with and without complications. Table 2 lists cases by specialty. Otorhinolaryngology (ENT) had the most numerous cases (*n* = 769, 45%) followed by medical imaging (*n* = 214, 12%). Fifty-one patients (2.92%) had a complication, as defined by the study metrics. Table 3 and Table 4 list the complication rate by specialty and complication type, respectively.

### 3.1. Respiratory Complications

In our data, respiratory events were the most common complication (*n* = 22, 43%). Of the respiratory events, hypoxemia was the most common (*n* = 12, 0.7%) and typically resolved within hours to days. Only one of the patients with hypoxemia (0.06%) also displayed severe stridor requiring several doses of racemic epinephrine and steroids. Two patients (0.12%) required reintubation after surgery for respiratory failure. Four patients (0.23%) had a new initiation of noninvasive positive pressure ventilation (CPAP or BiPAP that was continued on discharge). Two patients (0.12%) remained intubated after surgery for airway protection due to airway narrowing and/or edema. Two patients (0.12%) presented to the Emergency Department with hypoxemia and respiratory distress after being discharged home. None of the respiratory events occurred in anesthetics for diagnostic imaging.

### 3.2. Gastrointestinal Complications

Twelve patients (0.7%) had poor oral intake leading to delayed discharge from the hospital. Two patients (0.12%) had delayed return of bowel function, one of whom had to be initiated on total parenteral nutrition.

### 3.3. Surgical Complications

Three patients (0.18%) required a second operation for hemorrhage. Two patients (0.12%) had hematemesis or bleeding needing admission and monitoring. One patient (0.06%) had new dysphagia and aspiration as a consequence of the surgery. One patient (0.06%) had a wound infection. One patient (0.06%) had surgical findings that warranted intensive care unit admission for monitoring.

### 3.4. Cardiovascular Complications

One patient (0.06%) experienced persistent bradyarrhythmia that required admission and monitoring. There were two patients (0.12%) with intraoperative cardiac arrests, both of whom had a return of spontaneous circulation. There were no reports of perioperative deaths in our data. Additionally, one patient (0.06%) was diagnosed intraoperatively with coarctation of the aorta necessitating admission and workup.

### 3.5. Neurologic

Two patients (0.12%) with a history of seizures had perioperative status epilepticus requiring intervention.

## 4. Discussion

Children with DS often have comorbid conditions [46,47] requiring multiple anesthetics in their lifetime. While the known airway, cardiac, pulmonary, and neuromuscular anomalies of DS might be expected to place these patients at higher risk for perioperative events, our data did not demonstrate this. At our institution, the perioperative complication rate for non-cardiac surgery or medical imaging was 2.98% for children with DS. There is no standardized definition of perioperative complications in the general pediatric literature and reported complication rates vary widely based on these differences in defining criteria, anywhere from 0.3% to nearly 50% [31,48,49]. Our complication rate is comparable to previously reported rates at institutions with dedicated pediatric anesthesiology providers taking care of complex or sicker patients (4.4% and 3.3%) [50,51]. There are studies demonstrating that children with DS are not at additional risk of perioperative complications in both cardiac and non-cardiac surgery [36,37,52,53,54,55]. However, there are certain surgeries where their outcomes are notably worse [24].

Perioperative respiratory events are one of the major causes of morbidity and mortality in pediatric patients undergoing anesthesia [31,32,49]. In our data, respiratory events were the most common complication (43.1%). Of the respiratory events, hypoxemia was the most common and typically resolved within hours to days. Several patients had more critical events such as needing reintubation postoperatively or readmission for respiratory failure. Complications reported in Borland et al. [35] (bradycardia, obstruction, difficult airway, post intubation croup, and bronchospasm) did not figure prominently in our data. This difference in the types of complications may be attributed to the advancement of pediatric anesthesia practice with the routine use of low-pressure cuffed endotracheal tubes and the increased utilization of supraglottic airway devices.

Inadequate oral intake was the second most common complication in our dataset (24%). The refusal to eat or drink postoperatively is multifactorial. It may be due to oropharyngeal pain and dysphagia, as in after airway surgery, and compounded by intellectual disability. While inadequate oral intake may not pose immediate life-threatening risks, it impacts postoperative recovery and patient well-being and may lead to unplanned admissions, lengthened hospital stays, and increased costs.

There was a notable difference in complication rates between outpatients and inpatients (3.4% vs. 1.0%) and 3.38 increased odds of a perioperative adverse event (*p* = 0.042, Table 5). This is a surprising finding as previous studies have demonstrated that outpatient surgery is safe in pediatric patients [56,57]. This discrepancy may be due to the relatively few number of inpatient surgeries (*n* = 293) or the additional time available to optimize inpatients preoperatively compared to outpatients. This may also represent an opportunity to enhance our outpatient preoperative medical optimization processes.

There was also a large variability in complication rates between specialties. For instance, orthopedic and pediatric surgery had much higher complication rates than the overall group (15.6% and 9.3%, respectively). And on both univariable and multivariate logistic regression, orthopedic surgical cases had a significantly increased odds ratio of suffering a perioperative adverse event (3.65 [1.07–10.9], *p* = 0.11; Table 6). Diagnostic medical imaging was the second most common anesthetic encounter with a significantly lower odds ratio of a perioperative adverse event (0.12 [0.01–0.55], *p* = 0.034; Table 6), and only one complication (0.5%). Even so, the single complication was severe and life-threatening brady-arrhythmia, highlighting that anesthesia for low-risk procedures is not without risk.

A significant finding in our data was that an increasing number of procedures, procedural complexity, and total intraoperative minutes was associated with perioperative complications. Our finding is consistent with that previously reported in the literature [58,59]. Sometimes efforts are made by providers to combine multiple procedures into a single anesthetic to reduce cost, parental time burdens, to limit anesthetic exposures, and to limit healthcare costs. Yet, one must balance the potential risk of a perioperative complication following a long anesthetic with the theoretical cost savings of combining procedures.

Patient age, sex, weight, and case urgency were not associated with higher complication rates, which was unexpected given the existing literature suggesting higher risks for severe cardiovascular and respiratory complications in neonates, infants, and patients with higher ASA score [34,60,61]. Several factors may explain this incongruity. First, we excluded high-risk procedures such as cardiac surgery and cardiac catheterization, which are associated with higher morbidity and mortality. Second, the complication rate observed in the present study likely underestimates the true incidence of events because it relied on self-reported data, which tend to underreport adverse clinical events when compared with automated data collection by anesthesia information management systems (AIMS) or an EMR [62]. In children, perioperative adverse events such as hypotension, desaturation, bradycardia, and laryngospasm are relatively common and often regarded as “normal” occurrences associated with to pediatric anesthesia. These events are often considered uneventful as they are easily managed with no sequelae. Finally, the experience of anesthesia providers plays a crucial role in patient safety [31,63]. These cases were performed at tertiary pediatric hospital by experienced pediatric anesthesiologists and their expertise may have mitigated risk.

This study has several limitations. There are potential biases and limitations inherent to a retrospective single center study due to center practices that can disproportionately affect outcomes. Small sample sizes for certain specialties limit the generalizability of the data. Each encounter was considered as a unique event; however, some patients received multiple anesthetics during the study period. In longitudinal data collection, cofactors such as age and weight will change from one encounter to the next, while other intrinsic patient factors will not. Further, there are no matched cohort data for children without DS undergoing non-cardiac surgery or diagnostic imaging. Also, modifiable patient factors which affect perioperative outcomes were not captured well in the EMR (i.e., recent upper respiratory tract infection, asthma, smoking/passive smoking) [2].

Reducing perioperative adverse events requires a multifaceted approach beginning with the identification of at-risk patients. This allows for effective planning, resource allocation, and decisions regarding the location of surgery, staffing, and postoperative disposition [64,65]. A thorough preoperative evaluation, including medical history, physical examination, and clinical assessment, should be completed. Using risk prediction tools can further guide clinical decisions [66,67,68,69]. While some risk factors, such as age, physical status, and type of surgery, are unmodifiable, several modifiable risk factors can reduce the incidence of perioperative adverse events. These include preoperative optimization of asthma, delaying elective surgery following recent respiratory illness, and addressing abnormal lab findings [31,48,49,70]. Our study found that certain specialties were associated with an elevated risk of perioperative adverse events, and that outpatients faced a higher risk overall. This highlights a potential opportunity to improve outpatient preoperative medical optimization at our center.

Intraoperative anesthetic management, including choices between general and regional anesthesia, neuromuscular blockade, airway management (supraglottic airway vs. endotracheal tube), and ventilation strategy, also play an important role in outcomes [31,48,64,71,72]. With the high incidence of obesity and obstructive sleep apnea in children with DS, judicious titration of opioids and the use of multimodal analgesia is recommended [30,64]. And though it is not feasible to have all pediatric cases managed by pediatric anesthesiologists, it is important to consider the experience of the anesthesia provider particularly for high-risk surgeries in this vulnerable group. Studies have shown that both provider experience and pediatric case volume affect the rate of severe critical events [31,32,63]. The type of center (community hospital vs. free-standing children’s hospital) does not appear to affect the incidence of perioperative adverse events. However, children with DS and other high-risk conditions should receive appropriate postoperative monitoring and be cared for at a center capable of providing an elevated level of care if needed [31,64,65,71]. While it is impossible to eliminate all perioperative adverse events, comprehensive preoperative preparation and thoughtful anesthetic management can reduce their frequency and severity.

## 5. Conclusions

In line with previous reports, children with DS appear to tolerate anesthesia well for non-cardiac surgery or diagnostic imaging. This is significant as our study is the first to examine a large number of ENT and diagnostic imaging cases—two of the most common reasons for children with DS to receive anesthesia. Further research is needed to explore the relationship between complications and perioperative variables, such as the type of surgery, prior respiratory history, or preexisting conditions, to better understand the true risks associated with anesthesia and surgery in children with DS. By better characterizing the perioperative risk profile for this group, our findings may help inform anesthetic management strategies, improve perioperative care, and ultimately enhance safety and outcomes for children with DS surgery or medical imaging.

## Figures and Tables

**Table 1:** Characteristics of patients with and without complications.

	No Complication *N* = 1662	Complication *N* = 51	*p* Value
Age, years (std dev)	6.58 (5.12)	6.43 (5.28)	0.85
Sex			0.26
F	669 (40.3%)	16 (31.4%)	
M	993 (59.7%)	35 (68.6%)	
Weight, kg (std dev)	23.8 (18.2)	22.6 (18.9)	0.65
Intraoperative Minutes	81.0 [50.0,126] ^†^	101 [77.5, 180]	<0.001
Total procedures	1.00 [1.00, 2.00]	2.00 [1.00, 3.00]	0.011
Total RVU ^‡^	3.52 [2.01, 6.91]	7.07 [4.36, 14.6]	<0.001
Case Type			0.59
Elective	1,537 (92.5%)	46 (90.2%)	
Non-elective	125 (7.52%)	5 (9.8%)	
ASA § physical status Classification			0.81
1	16 (0.96%)	0 (0.00%)	
2	529 (31.9%)	14 (27.5%)	
3	1053 (63.5%)	36 (70.6%)	
4–5	61 (3.68%)	1 (2.0%)	
Admission status			0.05
Inpatient	290 (17.4%)	3 (5.9%)	
Outpatient	1372 (82.6%)	48 (94.1%)	

^†^ Median [first interquartile range, third interquartile range], ^‡^ RVU, relative value units, ^§^ ASA, American Society of Anesthesiologists.

**Table 2:** Cases by specialty.

Specialty	N (%), Total *N* = 1713
Anesthesiology	1 (<0.1%)
Audiology	34 (2.0%)
Dentistry	88 (5.1%)
Dermatology	1 (<0.1%)
Otorhinolaryngology	769 (45%)
Gastroenterology	59 (3.4%)
Hematology/oncology	45 (2.6%)
Interventional radiology	120 (7.0%)
Medical imaging	214 (12%)
Neurosurgery	23 (1.3%)
Ophthalmology	96 (5.6%)
Oral and maxillofacial surgery	3 (0.2%)
Orthopedic surgery	37 (2.2%)
Pediatric surgery	159 (9.3%)
Plastic surgery	14 (0.8%)
Pulmonary	2 (0.1%)
Rheumatology	3 (0.2%)
Transplant	3 (0.2%)
Urology	42 (2.5%)

**Table 3:** Complication Rate by Specialty.

Specialty	No Complication, *n*	Complication, *n*	Complication Rate
Otorhinolaryngology	738	31	4.2%
Medical imaging	213	1	0.5%
Pediatric surgery	151	8	5.3%
Interventional radiology	119	1	0.8%
Ophthalmology	95	1	1.1%
Dentistry	87	1	1.1%
Gastroenterology	57	2	3.5%
Hematology/oncology	45	0	0.0%
Urology	42	0	0.0%
Orthopedic surgery	32	5	15.6%
Audiology	34	0	0.0%
Neurosurgery	22	1	4.5%
Plastic Surgery	14	0	0.0%
Oral and maxillofacial surgery	3	0	0.0%
Rheumatology	3	0	0.0%
Transplant surgery	3	0	0.0%
Pulmonary	2	0	0.0%
Anesthesiology	1	0	0.0%
Dermatology	1	0	0.0%

**Table 4:** Complication Types.

Complication Type	*n* = 51	
Respiratory	22	43.1%
Gastrointestinal	15	29.4%
Surgical	8	15.7%
Cardiovascular	4	7.8%
Neurologic	2	3.9%

**Table 5:** Univariate logistic regression model predicting complication.

Characteristic	OR ^1^ [95% CI ^2^]	*p*-Value
Age	1.0 [0.94–1.05]	0.845
Sex		
F	-	
M	1.47 [0.82–2.75]	0.205
Weight	1.00 [0.98–1.01]	0.639
Procedural time	1.00 [1.00–1.01]	0.000
Total procedures	1.27 [1.00–1.59]	0.039
Total RVU ^3^	1.07 [1.04–1.09]	0.000
Case urgency		
Elective	-	
Non elective	1.34 [0.46–3.12]	0.546
ASA		
4/5	-	
1	0.00 [0.00–78,898]	0.982
2	1.61 [0.32–29.5]	0.646
3	2.09 [0.44–37.4]	0.472
Admission status		
Inpatient	-	
Outpatient	3.38 [1.23–14.0]	0.042
Specialty		
Otorhinolaryngology	-	
Dentistry	0.27 [0.02–1.30]	0.205
Gastroenterology	0.84 [0.13–2.86]	0.808
Interventional radiology	0.20 [0.01–0.95]	0.115
Medical Imaging	0.11 [0.01–0.52]	0.032
Neurosurgery	1.08 [0.06–5.42]	0.939
Ophthalmology	0.25 [0.01–1.19]	0.176
Orthopedics	3.72 [1.21–9.47]	0.011
Pediatric surgery	1.26 [0.52–2.67]	0.568
Other	0 [0–2,254,990,197]	0.985

^1^ OR, Odds ratio. ^2^ CI, confidence interval. ^3^ RVU, relative value units.

**Table 6:** Multivariate logistic regression model predicting complications.

Characteristic	OR ^1^ [95% CI ^2^]	*p*-Value
Age	1.00 [0.93–1.06]	>0.9
Sex		
F	-	
M	1.47 [0.81–2.77]	0.2
Specialty		
Otorhinolaryngology	-	
Dentistry	0.27 [0.02–1.34]	0.2
Gastroenterology	0.88 [0.14–3.06]	0.9
Interventional radiology	0.20 [0.01–0.96]	0.12
Medical imaging	0.12 [0.01–0.55]	0.034
Neurosurgery	1.19 [0.06–6.23]	0.9
Ophthalmology	0.25 [0.01–1.20]	0.2
Orthopedics	3.65 [1.07–10.9]	0.026
Pediatric surgery	1.26 [0.53–2.68]	0.6
Other	0.00 [0–2,010,040,591]	>0.9

^1^ OR, Odds ratio, ^2^ CI, confidence interval.

## Data Availability

The data that support the findings of this study are available from Ann and Robert H. Lurie Children’s Hospital of Chicago. Restrictions apply to the availability of these data, which were used under license for this study. Data are available from the authors with the permission of Ann and Robert H. Lurie Children’s Hospital of Chicago.

## References

[1] de Graaf G., Buckley F., Skotko B.G. (2015). Estimates of the live births, natural losses, and elective terminations with Down syndrome in the United States. Am. J. Med. Genet. A.

[2] Lejeune J., Gautier M., Turpin R. (1959). Etude des chromosomes somatiques de neuf enfants mongoliens. Comptes Rendus Hebd. Séances L’académie Sci..

[3] Penrose L.S. (1949). The incidence of mongolism in the general population. J. Ment. Sci..

[4] Collmann R.D., Stoller A. (1963). A life table for mongols in Victoria, Australia. J. Ment. Defic. Res..

[5] Leonard S., Bower C., Petterson B., Leonard H. (2000). Survival of infants born with Down’s syndrome: 1980-96. Paediatr. Perinat. Epidemiol..

[6] de Graaf G., Buckley F., Skotko B.G. (2017). Estimation of the number of people with Down syndrome in the United States. Genet. Med..

[7] Glasson E.J., Sullivan S.G., Hussain R., Petterson B.A., Montgomery P.D., Bittles A.H. (2002). The changing survival profile of people with Down’s syndrome: Implications for genetic counselling. Clin. Genet..

[8] Glasson E.J., Dye D.E., Bittles A.H. (2014). The triple challenges associated with age-related comorbidities in Down syndrome. J. Intellect. Disabil. Res..

[9] de Jong A.L., Sulek M., Nihill M., Duncan N.O., Friedman E.M. (1997). Tenuous airway in children with trisomy 21. Laryngoscope.

[10] Nakazawa K., Ikeda D., Ishikawa S., Makita K. (2003). A case of difficult airway due to lingual tonsillar hypertrophy in a patient with Down’s syndrome. Anesth. Analg..

[11] Belanger J., Kossick M. (2015). Methods of identifying and managing the difficult airway in the pediatric population. AANA J..

[12] Bull M.J. (2020). Down Syndrome. N. Engl. J. Med..

[13] Donnelly L.F., Shott S.R., LaRose C.R., Chini B.A., Amin R.S. (2004). Causes of persistent obstructive sleep apnea despite previous tonsillectomy and adenoidectomy in children with down syndrome as depicted on static and dynamic cine MRI. AJR Am. J. Roentgenol..

[14] Watts R., Vyas H. (2013). An overview of respiratory problems in children with Down’s syndrome. Arch. Dis. Child..

[15] Hamilton J., Yaneza M.M., Clement W.A., Kubba H. (2016). The prevalence of airway problems in children with Down’s syndrome. Int. J. Pediatr. Otorhinolaryngol..

[16] Miller R., Gray S.D., Cotton R.T., Myer C.M., Netterville J. (1990). Subglottic stenosis and Down syndrome. Am. J. Otolaryngol..

[17] Martinez-Espinosa R.M., Molina Vila M.D., Reig Garcia-Galbis M. (2020). Evidences from Clinical Trials in Down Syndrome: Diet, Exercise and Body Composition. Int. J. Environ. Res. Public Health.

[18] Bertapelli F., Pitetti K., Agiovlasitis S., Guerra-Junior G. (2016). Overweight and obesity in children and adolescents with Down syndrome-prevalence, determinants, consequences, and interventions: A literature review. Res. Dev. Disabil..

[19] Jacobs I.N., Gray R.F., Todd N.W. (1996). Upper airway obstruction in children with Down syndrome. Arch. Otolaryngol. Head. Neck Surg..

[20] Pandit C., Fitzgerald D.A. (2012). Respiratory problems in children with Down syndrome. J. Paediatr. Child. Health.

[21] Lewanda A.F., Matisoff A., Revenis M., Harahsheh A., Futterman C., Nino G., Greenberg J., Myseros J.S., Rosenbaum K.N., Summar M. (2016). Preoperative evaluation and comprehensive risk assessment for children with Down syndrome. Paediatr. Anaesth..

[22] Stoll C., Dott B., Alembik Y., Roth M.P. (2015). Associated congenital anomalies among cases with Down syndrome. Eur. J. Med. Genet..

[23] Irving C.A., Chaudhari M.P. (2012). Cardiovascular abnormalities in Down’s syndrome: Spectrum, management and survival over 22 years. Arch. Dis. Child..

[24] Dimopoulos K., Constantine A., Clift P., Condliffe R., Moledina S., Jansen K., Inuzuka R., Veldtman G.R., Cua C.L., Tay E.L.W. (2023). Cardiovascular Complications of Down Syndrome: Scoping Review and Expert Consensus. Circulation.

[25] Bai W., Voepel-Lewis T., Malviya S. (2010). Hemodynamic changes in children with Down syndrome during and following inhalation induction of anesthesia with sevoflurane. J. Clin. Anesth..

[26] Kraemer F.W., Stricker P.A., Gurnaney H.G., McClung H., Meador M.R., Sussman E., Burgess B.J., Ciampa B., Mendelsohn J., Rehman M.A. (2010). Bradycardia during induction of anesthesia with sevoflurane in children with Down syndrome. Anesth. Analg..

[27] Lagan N., Huggard D., Mc Grane F., Leahy T.R., Franklin O., Roche E., Webb D., O’Marcaigh A., Cox D., El-Khuffash A. (2020). Multiorgan involvement and management in children with Down syndrome. Acta Paediatr..

[28] McDowell K.M., Craven D.I. (2011). Pulmonary complications of Down syndrome during childhood. J. Pediatr..

[29] Ramba M., Bogunovic D. (2024). The immune system in Down Syndrome: Autoimmunity and severe infections. Immunol. Rev..

[30] Egbuta C., Mason K.P. (2020). Recognizing Risks and Optimizing Perioperative Care to Reduce Respiratory Complications in the Pediatric Patient. J. Clin. Med..

[31] Habre W., Disma N., Virag K., Becke K., Hansen T.G., Johr M., Leva B., Morton N.S., Vermeulen P.M., Zielinska M. (2017). Incidence of severe critical events in paediatric anaesthesia (APRICOT): A prospective multicentre observational study in 261 hospitals in Europe. Lancet Respir. Med..

[32] Mamie C., Habre W., Delhumeau C., Argiroffo C.B., Morabia A. (2004). Incidence and risk factors of perioperative respiratory adverse events in children undergoing elective surgery. Paediatr. Anaesth..

[33] Bhananker S.M., Ramamoorthy C., Geiduschek J.M., Posner K.L., Domino K.B., Haberkern C.M., Campos J.S., Morray J.P. (2007). Anesthesia-related cardiac arrest in children: Update from the Pediatric Perioperative Cardiac Arrest Registry. Anesth. Analg..

[34] Disma N., Veyckemans F., Virag K., Hansen T.G., Becke K., Harlet P., Vutskits L., Walker S.M., de Graaff J.C., Zielinska M. (2021). Morbidity and mortality after anaesthesia in early life: Results of the European prospective multicentre observational study, neonate and children audit of anaesthesia practice in Europe (NECTARINE). Br. J. Anaesth..

[35] Borland L.M., Colligan J., Brandom B.W. (2004). Frequency of anesthesia-related complications in children with Down syndrome under general anesthesia for noncardiac procedures. Paediatr. Anaesth..

[36] Cairo S.B., Zeinali L.I., Berkelhamer S.K., Harmon C.M., Rao S.O., Rothstein D.H. (2019). Down Syndrome and Postoperative Complications in Children Undergoing Intestinal Operations. J. Pediatr. Surg..

[37] Evans J.M., Dharmar M., Meierhenry E., Marcin J.P., Raff G.W. (2014). Association between Down syndrome and in-hospital death among children undergoing surgery for congenital heart disease: A US population-based study. Circ. Cardiovasc. Qual. Outcomes.

[38] Graber T.J., Baskin P.L., Soria C., Greenberg M., Gabriel R.A., Brzenski A. (2021). An assessment of perioperative respiratory adverse events and difficult intubation in pediatric patients with Trisomy 21. Paediatr. Anaesth..

[39] Yumusakhuylu A.C., Binnetoglu A., Demir B., Baglam T., Sari M. (2016). Is it safe to perform adenotonsillectomy in children with Down syndrome?. Eur. Arch. Otorhinolaryngol..

[40] Sha S., Abdelsabour H., Vijimohan S.J., Board T., Alshryda S. (2019). Total hip arthroplasty in patients with Trisomy 21: Systematic review and exploratory patient level analysis. Surgeon.

[41] Bartels D.D., McCann M.E., Davidson A.J., Polaner D.M., Whitlock E.L., Bateman B.T. (2018). Estimating pediatric general anesthesia exposure: Quantifying duration and risk. Paediatr. Anaesth..

[42] Gloff M.S., Robinson R., Correll L.R., Lander H., Pyne S., Webber A. (2022). Preoperative optimization in the pediatric patient. Int. Anesthesiol. Clin..

[43] Lerman J. (2019). Pediatric ambulatory anesthesia: An update. Curr. Opin. Anaesthesiol..

[44] Construction Financial Management Association National Physician Fee Schedule Relative Value File January Release. https://www.cms.gov/medicare/payment/fee-schedules/physician/pfs-relative-value-files.

[45] Dyas A.R., Meguid R.A., Bronsert M.R., Madsen H.J., Colborn K.L., Lambert-Kerzner A., Henderson W.G. (2023). Does Work Relative Value Unit Measure Surgical Complexity for Risk Adjustment of Surgical Outcomes?. J. Surg. Res..

[46] Alexander M., Petri H., Ding Y., Wandel C., Khwaja O., Foskett N. (2016). Morbidity and medication in a large population of individuals with Down syndrome compared to the general population. Dev. Med. Child. Neurol..

[47] Roizen N.J., Magyar C.I., Kuschner E.S., Sulkes S.B., Druschel C., van Wijngaarden E., Rodgers L., Diehl A., Lowry R., Hyman S.L. (2014). A community cross-sectional survey of medical problems in 440 children with Down syndrome in New York State. J. Pediatr..

[48] von Ungern-Sternberg B.S., Boda K., Chambers N.A., Rebmann C., Johnson C., Sly P.D., Habre W. (2010). Risk assessment for respiratory complications in paediatric anaesthesia: A prospective cohort study. Lancet.

[49] von Ungern-Sternberg B.S., Sommerfield D., Slevin L., Drake-Brockman T.F.E., Zhang G., Hall G.L. (2019). Effect of Albuterol Premedication vs Placebo on the Occurrence of Respiratory Adverse Events in Children Undergoing Tonsillectomies: The REACT Randomized Clinical Trial. JAMA Pediatr..

[50] Engelhardt T., Ayansina D., Bell G.T., Oshan V., Rutherford J.S., Morton N.S., APRICOT Group of the European Society of Anaesthesiology Clinical Trial Network (2019). Incidence of severe critical events in paediatric anaesthesia in the United Kingdom: Secondary analysis of the anaesthesia practice in children observational trial (APRICOT study). Anaesthesia.

[51] Hansen T.G., Borke W.B., Isohanni M.H., Castellheim A., APRICOT Study Group of the European Society of Anaesthesiology Clinical Trial Network (2019). Incidence of severe critical events in paediatric anaesthesia in Scandinavia: Secondary analysis of Anaesthesia PRactice in Children Observational Trial (APRICOT). Acta Anaesthesiol. Scand..

[52] Toth R., Szanto P., Prodan Z., Lex D.J., Sapi E., Szatmari A., Gal J., Szanto T., Szekely A. (2013). Down syndrome and postoperative complications after paediatric cardiac surgery: A propensity-matched analysis. Interact. Cardiovasc. Thorac. Surg..

[53] Hoashi T., Hirahara N., Murakami A., Hirata Y., Ichikawa H., Kobayashi J., Takamoto S. (2018). Current Surgical Outcomes of Congenital Heart Surgery for Patients with Down Syndrome in Japan. Circ. J..

[54] St Louis J.D., Jodhka U., Jacobs J.P., He X., Hill K.D., Pasquali S.K., Jacobs M.L. (2014). Contemporary outcomes of complete atrioventricular septal defect repair: Analysis of the Society of Thoracic Surgeons Congenital Heart Surgery Database. J. Thorac. Cardiovasc. Surg..

[55] Bartz-Kurycki M.A., Anderson K.T., Austin M.T., Kao L.S., Tsao K., Lally K.P., Kawaguchi A.L. (2018). Increased complications in pediatric surgery are associated with comorbidities and not with Down syndrome itself. J. Surg. Res..

[56] Crute W., Wofford A., Powers J., Smith D.P. (2023). Comprehensive review of a large cohort of outpatient versus inpatient open renal and bladder surgery in children. J. Pediatr. Urol..

[57] Jardaly A., Torrez T.W., McGwin G., Gilbert S.R. (2022). Comparing complications of outpatient management of slipped capital femoral epiphysis and Blount’s disease: A database study. World J. Orthop..

[58] Miketic R.M., Uffman J., Tumin D., Tobias J.D., Raman V.T. (2019). Experience with Combining Pediatric Procedures into a Single Anesthetic. Pediatr. Qual. Saf..

[59] Cheon E.C., Palac H.L., Paik K.H., Hajduk J., De Oliveira G.S., Jagannathan N., Suresh S. (2016). Unplanned, Postoperative Intubation in Pediatric Surgical Patients: Development and Validation of a Multivariable Prediction Model. Anesthesiology.

[60] van der Griend B.F., Lister N.A., McKenzie I.M., Martin N., Ragg P.G., Sheppard S.J., Davidson A.J. (2011). Postoperative mortality in children after 101,885 anesthetics at a tertiary pediatric hospital. Anesth. Analg..

[61] Murat I., Constant I., Maud’huy H. (2004). Perioperative anaesthetic morbidity in children: A database of 24,165 anaesthetics over a 30-month period. Paediatr. Anaesth..

[62] Simpao A.F., Pruitt E.Y., Cook-Sather S.D., Gurnaney H.G., Rehman M.A. (2012). The reliability of manual reporting of clinical events in an anesthesia information management system (AIMS). J. Clin. Monit. Comput..

[63] Zgleszewski S.E., Graham D.A., Hickey P.R., Brustowicz R.M., Odegard K.C., Koka R., Seefelder C., Navedo A.T., Randolph A.G. (2016). Anesthesiologist- and System-Related Risk Factors for Risk-Adjusted Pediatric Anesthesia-Related Cardiac Arrest. Anesth. Analg..

[64] Brennan M.P., Webber A.M., Patel C.V., Chin W.A., Butz S.F., Rajan N. (2024). Care of the Pediatric Patient for Ambulatory Tonsillectomy with or Without Adenoidectomy: The Society for Ambulatory Anesthesia Position Statement. Anesth. Analg..

[65] Templeton T.W., Sommerfield D., Hii J., Sommerfield A., Matava C.T., von Ungern-Sternberg B.S. (2022). Risk assessment and optimization strategies to reduce perioperative respiratory adverse events in Pediatric Anesthesia-Part 2: Anesthesia-related risk and treatment options. Paediatr. Anaesth..

[66] Nasr V.G., DiNardo J.A., Faraoni D. (2017). Development of a Pediatric Risk Assessment Score to Predict Perioperative Mortality in Children Undergoing Noncardiac Surgery. Anesth. Analg..

[67] Tait A.R., Voepel-Lewis T., Christensen R., O’Brien L.M. (2013). The STBUR questionnaire for predicting perioperative respiratory adverse events in children at risk for sleep-disordered breathing. Paediatr. Anaesth..

[68] Subramanyam R., Yeramaneni S., Hossain M.M., Anneken A.M., Varughese A.M. (2016). Perioperative Respiratory Adverse Events in Pediatric Ambulatory Anesthesia: Development and Validation of a Risk Prediction Tool. Anesth. Analg..

[69] Lee L.K., Bernardo M.K.L., Grogan T.R., Elashoff D.A., Ren W.H.P. (2018). Perioperative respiratory adverse event risk assessment in children with upper respiratory tract infection: Validation of the COLDS score. Paediatr. Anaesth..

[70] Hii J., Templeton T.W., Sommerfield D., Sommerfield A., Matava C.T., von Ungern-Sternberg B.S. (2022). Risk assessment and optimization strategies to reduce perioperative respiratory adverse events in pediatric anesthesia-Part 1 patient and surgical factors. Paediatr. Anaesth..

[71] August D.A., Everett L.L. (2014). Pediatric ambulatory anesthesia. Anesthesiol. Clin..

[72] Drake-Brockman T.F., Ramgolam A., Zhang G., Hall G.L., von Ungern-Sternberg B.S. (2017). The effect of endotracheal tubes versus laryngeal mask airways on perioperative respiratory adverse events in infants: A randomised controlled trial. Lancet.

